# Circulating exosomal microRNAs as biomarkers of lupus nephritis

**DOI:** 10.3389/fimmu.2023.1326836

**Published:** 2023-12-29

**Authors:** Fei Chen, Bo Shi, Wenjing Liu, Jianmin Gong, Jia Gao, Yifan Sun, Ping Yang

**Affiliations:** ^1^ Department of Clinical Laboratory, The Affiliated Drum Tower Hospital of Nanjing University Medical School, Nanjing, China; ^2^ College of Life Sciences, Yangtze University, Jingzhou, China; ^3^ Department of Orthopedics, The Second Affiliated Hospital of Nanjing Medical University, Nanjing, China; ^4^ Department of Laboratory Medicine, Nanjing Drum Tower Hospital Clinical College of Jiangsu University, Nanjing, China

**Keywords:** systemic lupus erythematosus, lupus nephritis, exosomes, miRNA, biomarkers

## Abstract

**Objective:**

Disruption in the delicate symphony of genes, microRNA (miRNA), or protein expression can result in the dysregulation of the immune system, leading to the devastating consequences such as lupus nephritis (LN). The capacity of exosomes to transport miRNAs between cells and modify the phenotype of recipient cells implies their involvement in persistent kidney inflammation. This study unveils identifying two previously undiscovered exosomal miRNAs in the serum of LN patients, offering potential solutions to the current challenges in LN diagnosis and management.

**Methods:**

Initially, we used a reagent-based kit to isolate serum exosomes from patients with Systemic lupus erythematosus (SLE) and used Trizol method for total RNA extraction. Subsequently, we employed small RNA sequencing to screen for differential expression profiles of exosomal small RNAs. The RT-qPCR method was used to individually validate samples in both the screening and validation cohorts, enabling the identification of candidate small RNAs; specific to LN. We assessed the diagnostic potency using receiver operating characteristic (ROC) curve, and explored the biological roles of miRNAs using Gene Ontology (GO) and Kyoto Encyclopedia of Genes and Genomes (KEGG) analyses.

**Results:**

Compared to SLE patients without LN, SLE patients accompanied by LN exhibited significantly spiked levels of exosomal hsa-miR-4796-5p and hsa-miR-7974. The duo of miRNAs, hsa-miR-4796-5p and hsa-miR-7974, exhibited promising potential as biomarkers for diagnosing LN, with an AUC exceeding 0.8. Correlation analysis revealed a strong positive association between these miRNAs and proteinuria, as well as the SLE Disease Activity Index (SLEDAI) score. Moreover, the levels of two miRNAs in LN patients were significantly elevated in comparison to other autoimmune nephritis conditions, such as immunoglobulin A nephropathy (IgAN) and diabetic nephropathy (DN). Furthermore, the bioinformatics analysis indicated that this miRNAs duo can play a pivotal role in the regulation of immune processes by modulating signal pathways, such as the mTOR and PI3K-Akt signaling pathway.

**Conclusion:**

This study provides a new ground that serum exosomal miRNAs can effectively identify and predict LN in SLE patients.

## Introduction

Systemic lupus erythematosus (SLE) continues to pose significant challenges within the realm of medicine, causing characteristic blend of systemic and organ-specific clinical manifestations, coupled with extensive dysfunction of the immune system (1, 2). LN stands out to be one of the most severe organic manifestations of SLE, affecting approximately 30-60% of adults and up to 70% of pediatric lupus patients (3). Furthermore, it is noteworthy that LN contributes significantly to the elevated incidence of SLE, heightened mortality rates, and increased healthcare expenditures (4). According to the guidelines, a reliable criterion for diagnosing LN is the histopathological confirmation obtained through renal biopsy (5, 6). However, kidney biopsy is an invasive procedure associated with the risk of bleeding and is not easily repeatable. Consequently, it poses limitations to rheumatology and immunology physicians in their ability to dynamically monitor and manage the disease progression of SLE. Currently, commonly used laboratory markers for LN include urinary protein, serum creatinine, glomerular filtration rate, anti-dsDNA antibody, and serum complements (7). However, these clinical parameters fall short of meeting the practical demands of clinical settings due to their insufficient sensitivity and specificity (8, 9). Therefore, it is crucial to discover novel non-invasive markers capable of detecting LN activity, predicting relapses, and monitoring treatment responses.

MicroRNAs (miRNAs), tiny non-coding RNAs (18-25 nucleotides) that regulate gene expression by binding to messenger RNA (mRNA), play a crucial role in cell biology and disease (10). This interaction prompts the restraint of mRNA translation and/or hastens its degradation, thus culminating in the curtailment of protein synthesis specific to certain target proteins (11, 12). In 2008, Chen Xi et al. (13) made the initial discovery of miRNA in human serum and provided evidence that it could serve as a new disease marker. In recent years, mounting evidence suggests the involvement of miRNA in the occurrence and development of various diseases, such as Alzheimer’s disease (AD), cancers, diabetes and autoimmune diseases (14–17). Extracellular vesicles(EVs) are membrane vesicles released by various cell types and can be categorized into three types: exosomes, microvesicles, and apoptotic bodies (18). Numerous studies have demonstrated that EVs play a significant role in the development of autoimmune diseases through various mechanisms (19). Microvesicles (MVs) are larger membrane vesicles derived from the cell plasma membrane surface. They can carry nuclear autoantigens and form immune complexes (ICs), which activate complements and cause damage to renal tissue (20). Nielsen CT et al. utilized immune electron microscopy technology to provide evidence of co-localization between glomerular deposited immune complexes (ICs) and microvesicles, as well as galectin-3-binding protein (G3BP) in LN (21). Exosomes, which are generated through the exocytosis of endosomal-derived intracellular membrane vesicles into the extracellular space (22), were initially discovered by Johnstone and colleagues in 1983 during the culture of reticulocytes (23). Previous studies have found that exosomes may contribute to a proinflammatory milieu and autoimmune inflammation in LN indirectly, either by directly interacting with their associated proinflammatory components or by triggering other cells to produce proinflammatory cytokines or materials (19, 24). The dysregulation of circulating exosomal miRNAs in autoimmune diseases has been extensively studied, and there is increasing evidence confirming their involvement (25, 26). However, the diagnostic potential of serum exosomal miRNAs in LN has not yet been fully explored.

In this study, our objective was to investigate the differential expression of miRNAs in exosomes derived from the serum of patients with LN. We employed RNA sequencing to screen for differential expression profiles of exosomal small RNAs. Subsequently, we identified the top ten upregulated miRNAs as potential candidate miRNAs. The RT-qPCR method was employed in both the screening and validation cohorts, facilitating the identification of candidate miRNAs specific to LN. To evaluate their potential as biomarkers, we conducted receiver-operator characteristic (ROC) curve analysis. Furthermore, we utilized bioinformatics tools including Gene Ontology (GO) and Kyoto Encyclopedia of Genes and Genomes (KEGG) analyses to predict the target genes of these miRNAs and explore their potential functions and associated pathways. Our meticulous analysis revealed that serum exosomal miRNAs can be utilized as non-invasive biomarkers for the identification and prediction of LN in individuals with SLE.

## Materials and methods

### Subjects and study design

**Figure 1:**
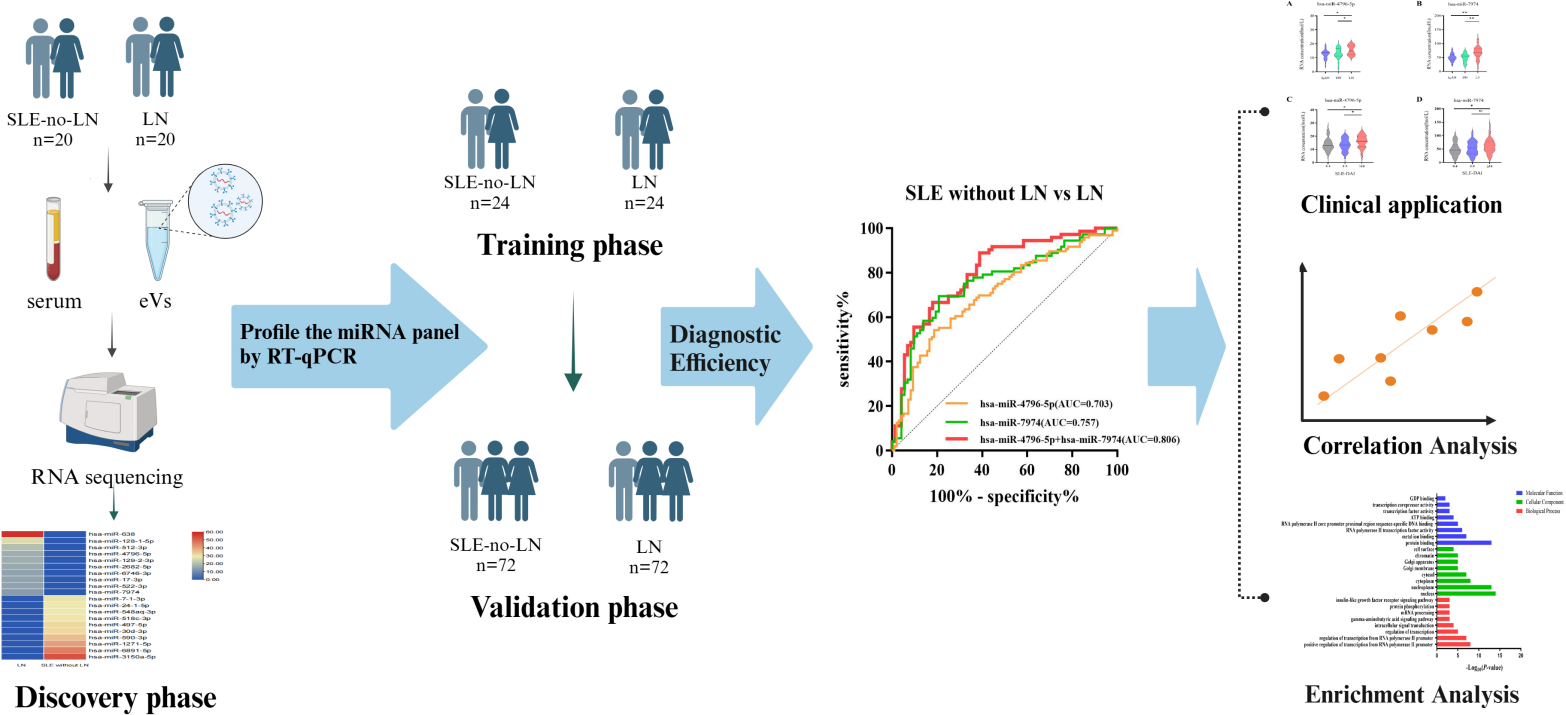
Workflow of the study.

This study enrolled 232 subjects from September 2022 to March 2023, including 116 patients with LN and 116 sex-age-matched patients with SLE without LN. All participants were hospitalized at Drum Tower of Nanjing Hospital and diagnosed by rheumatologists. The inclusion criteria for participants with SLE were based on the modified American College of Rheumatology 1997 revised criteria (27, 28). Individuals with a history of malignant tumors, concurrent infections, metabolic abnormalities, or concurrent other autoimmune diseases were excluded from the study cohort. The subjects were divided into three phases (**Figure 1**). During the discovery phase, we conducted miRNA sequencing by extracting exosomes from a 10 ml serum pool consisting of 20 LN patients and 20 SLE without LN patients (GEO number: GSE179950). In the training and validation phase, the expression levels of candidate miRNAs were validated in 192 samples using RT-qPCR assay. The activity of the disease was evaluated by the SLE Disease Activity Index (SLEDAI) (29). Approval to conduct this study was provided by the Ethics Committee of the Affiliated Drum Tower Hospital of Nanjing University Medical School, with the assigned approval identification number 2020-327-01. Prior to their participation in this experiment, all individuals involved provided written informed consent. The clinical and demographic characteristics of all participants are presented in detail in **Table 1**.

**Table 1:** Clinical and demographic characteristics of all participants.

Characteristics	SLE without LN (n=116)	LN (n=116)	*P* value
Age, years	40(29-51)	35(27-47)	0.158
Female, n (%)	97(89.81)	93(86.11)	0.365
Proteinuria, n (%)	34(31.48)	98(90.74)	<0.001***
Hematuria, n (%)	19(17.59)	75(69.44)	<0.001***
Pyuria, n (%)	27(25.00)	50(46.30)	0.003**
Cylinderuria, n (%)	0(0)	42(38.89)	<0.001***
24h proteinuria, median (IQR), mg/24h	137.0(67.0-201.0)	2069.0(699.5-5489.7)	<0.001***
ACR, median (IQR), mg/g	9.2(5.6-24.3)	789.3(189.9-2308.1)	<0.001***
ESR, median (IQR), mm/h	22(10-42)	32(16-57)	0.003**
Total protein, median (IQR), g/L	66.2(63.3-72.5)	55.0(48.8-64.3)	<0.001***
Blood albumin, median (IQR), g/L	37.9(35.4-40.6)	31.9(28.6-36.7)	<0.001***
Globulin, median (IQR), g/L	29.3(25.2-33.8)	22.7(18.4-28.6)	<0.001***
A/G, median (IQR)	1.29(1.12-1.54)	1.39(1.15-1.68)	0.056
GLU, median (IQR), mmol/L	4.46(4.11-4.84)	4.37(3.90-4.86)	0.111
Urea nitrogen, median (IQR), mmol/L	4.9(3.9-6.0)	7.4(5.2-11.5)	<0.001***
CREA, median (IQR), umol/L	49(41-56)	61(47-88)	<0.001***
Uric acid, median (IQR), umol/L	293(208-359)	391(296-452)	<0.001***
Total CO2, median (IQR), mmol/L	24.3(23.0-25.3)	23.6(21.1-25.2)	0.008**
eGFR, median (IQR), ml/min/1.73m^2	132.7(110.6-164.9)	103.5(75.2-145.0)	<0.001***
C1q, median (IQR), mg/dL	15.9(13.7-18.4)	13.2(11.2-17.0)	0.007**
C3, median (IQR), g/L	0.82(0.62-1.01)	0.76(0.47-0.96)	0.041*
C4, median (IQR), g/L	0.12(0.07-0.19)	0.13(0.05-0.19)	0.702
anti-dsDNA, median (IQR), IU/mL	87.43(18.40-253.71)	112.31(19.74-457.58)	0.392
ANA, n (%)	88(81.48)	86(79.63)	0.693
25-(OH) D3, median (IQR), ng/mL	18.50(13.36-24.29)	14.06(8.76-28.19)	0.051
SLE-DAI, median (IQR)	4(2-5)	12(8-15)	<0.001***

Abbreviations: ACR, albumin-to-creatinine ratio; ESR, erythrocyte sedimentation rate; GLU, glucose; CREA, serum creatinine; eGFR, glomerular filtration rate; C1q, complement 1q; C3, complement C3; C4, complement C4; anti-dsDNA, anti-double stranded DNA antibody; ANA, antinuclear antibodies; SLE-DAI, systemic lupus erythematosus disease activity index. *P < 0.05, **P < 0.01, ***P < 0.001 (Mann-Whitney U test).

### Library preparation and RNA sequencing

Prior to sequencing, the integrity of RNA samples was validated using agarose gel electrophoresis. The quantification of the RNA samples was achieved using the NanoDrop ND-1000 instrument. To account for the heavy decoration of miRNAs with RNA modifications, several treatments were conducted before library construction. These treatments included 3′-aminoacyl deacylation to 3′-OH for 3′-adaptor ligation, removal of 3′-cP to 3′-OH for 3′-adaptor ligation, 5′-OH phosphorylation to 5′-P for 5′-adaptor ligation, and demethylation of m1A and m3C. The library construction and deep sequencing were carried out by BGI (Shenzhen, China) using the Illumina Next Seq instrument. The sequencing libraries were optimized for RNA biotypes and validated using the Agilent 2100 Bioanalyzer. The sequencing process consisted of 50 cycles.

### Exosomes isolation

The serum samples were centrifuged at 2000 g for 30 min to separate cells and debris. Afterwards, they were stored at -80°C and thawed only when needed for use. Exosomes were isolated from serum using a commercial kit named as Total Exosome Isolation reagent (Thermo Fisher scientific, US) according to the manufacturer’s instructions. Briefly, a 100 μL serum sample was mixed with 20 μL of kit reagent and incubated at 4°C for 24 h. Then, the sample was centrifuged at 4°C at 10000 g for 10 min. Discarded the supernatant while the exosome pellets were resuspended in 100 μL of 1×phosphate buffer saline (PBS) for further analysis.

### Exosomes sizing

For measuring the size, exosomes were first diluted in 1 ml of PBS. The mixture was gently inverted to ensure even distribution and then slowly added to the NTA sample cell using a 1 ml disposable syringe. Three sample videos, each lasting 30 to 60 seconds, were recorded. The ZetaView Nanoparticle Tracking Analysis (NTA) system was utilized to measure the average diameter of the exosomes. This system has the capability to characterize the size distribution of small particles in liquid samples and can detect diameters ranging from 20 to 1000 nm (30). The results obtained from processing the Software ZetaView (Zeta View 8.04.02) were expressed as the mean standard deviation (SD) of the three video recordings.

### Transmission electron microscopy

A total of 20 μL of exosomes were applied onto the copper grid of the electron microscope and allowed to sit at room temperature for 10 min. Subsequently, 20 μL of 2% phosphotungstic acid was added to the copper grid for negative staining, which was carried out for 10 min. Excess staining was removed using filter paper. Once the copper grid was dried, transmission electron microscopy (TEM) was performed to examine the exosomes. The test was conducted under the at 120KV. A bilayer membrane structure was selected, and particles with diameters ranging from 100 to 200 nm were captured in the photographs.

### Western blotting

The exosomes obtained from serum were treated with a lysis solution containing RIPA lysis buffer. After 30 min of lysis on ice, the mixture was centrifuged at 4°C at 12000 g for 10 min. The resulting supernatant was collected, and the protein concentration in exosomes was measured using the Micro BCA protein detection kit (Thermo Fisher Scientific, California, USA). The remaining proteins were added to 5 × SDS loading buffer and heated at 99°C for 5 min. Subsequently, 20 μg of protein was loaded onto a 0.2 µm PVDF membrane following the manufacturer’s protocol. The loading process involved applying a voltage of 80 V for 30 min, followed by 120 V for 1 h. The membranes were then blocked with 5% skim milk at room temperature for 1 h and incubated overnight at 4°C with primary antibodies targeting CD63, TSG101, and Calnexin (Abcam, UK) (21). They were washed four times with 1× TBST for 15 minutes each and incubated with the appropriate secondary antibody for 1 h at room temperature. Finally, the membranes were washed three times with TBST for 10 min each, the ECL exposure solution was applied to the film, and pictures were taken.

### RNA extraction and RT-qPCR

Total RNAs were extracted from serum exosomes using the Trizol reagent (Invitrogen, USA) and were dissolved in water treated by diethylpyrocarbonate (DEPC). Briefly, Trizol is a phenol-guanidine isothiocyanate solution that effectively lyses biological material and denatures proteins (31). It is designed to preserve RNA integrity. After adding chloroform and separating the phases, proteins are extracted in the organic phase, DNA is separated at the interface, and RNA is left in the aqueous phase. The aqueous phase can be carefully aspirated and isopropanol can be added to precipitate the RNA. The content and purity of acquired RNA were detected by OneDrop-2000 (Nano Drop Technologies) miRNAs were reverse transcribed into cDNA using miRNA 1st Strand cDNA Synthesis Kit (Vazyme Biotech). The quantitative RT-qPCR reaction was performed in 96-well plates with the miRNA Universal SYBR qPCR Master Mix (Vazyme Biotech). The primer details for the 10 candidate miRNAs can be found in **Supplementary Table 1**.

### Pathway analysis

For a more in-depth insight into the function of the target genes of miRNAs, we conducted gene ontology (GO) and annotations Kyoto Encyclopedia of Genes and Genomes (KEGG). Predictions of miRNAs for LN were performed using the DAVID database (http://david.ncifcrf.gov/), which integrates biological data and analysis tools to offer detailed information on the functional annotations of genes/proteins. To be significant the *P*-value (*P* < 0.05) was considered.

### Statistical analysis

Continuous variables were represented as the median (interquartile range [IQR]) based on the normality test. The Spearman rank test was used to compare miRNA expression and clinical variables between two groups. Receiver operator characteristic (ROC) analysis was performed to calculate the area under the ROC curves (AUC), which evaluated the diagnostic efficiency of the candidate miRNAs. Statistical analyses were conducted using SPSS software, version 20.0 (SPSS Inc., Chicago, IL, USA), and GraphPad Prism version 9 (GraphPad Software Inc., La Jolla, CA, USA). The Mann-Whitney U test (*P < 0.05) was used to determine statistical significance. SPSS binary logistic regression was employed to predict the probability of joint diagnosis. The correlation analysis data were analyzed using Spearman rank correlation analysis.

### Data and materials availability

miRNA sequencing data, deposited in GEO under GSE179950. The raw data is available upon request from the corresponding author, providing a valuable resource for further research.

## Results

### Identification and characterization of serum exosomes

**Figure 2:**
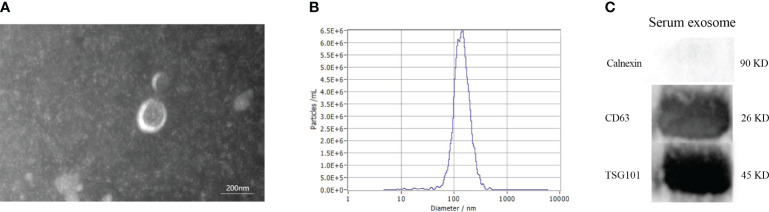
Characteristics of serum exosomes. **(A)** Morphology of vesicles under transmission electron microscopy. **(B)** Particle size distribution of vesicles measured by Nanoparticle Tracking Analysis. **(C)** Western blotting revealed the presence of exosomal biomarkers such as CD63, and TSG101.

The morphology of serum exosomes was verified using TEM and the particle size using NTA. The results showed the presence of elliptical or bowl-shaped particles with an average diameter of 127 nm (**Figures 2A, B**). Western blot analysis also revealed the presence of typical EV proteins such as CD63 and TSG101 and absence of calnexin (**Figure 2C**). These findings demonstrate the existence of exosomes in serum, providing a basis for further investigation.

### miRNA profiles of serum exosomes from LN patients

**Figure 3:**
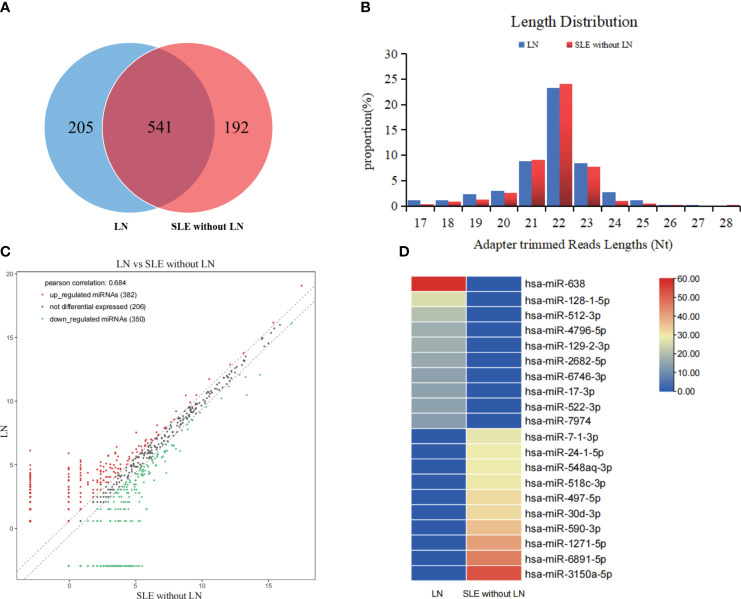
Analysis of differentially expressed miRNAs in serum exosomes of LN patients. **(A)** Venn diagram of serum exosomes derived miRNAs in LNs and SLE without LN patients. **(B)** The profiles of various length of miRNAs in serum exosomes between the two cohorts. **(C)** Scatter plots of differentially expressed miRNAs. Red and green dots indicated upregulated and downregulated miRNAs (log2 fold change > 2 between the two compared cohorts), and black dots indicated non-differentially expressed miRNAs. **(D)** Hierarchical clustering indicated the profiles of top 10 upregulated and downregulated miRNAs between two cohorts.

To determine the miRNA profile in LN patients, all participants were divided into two groups according to the clinical standard, including SLE with LN and SLE without LN. Exosomes were extracted from the mixed serum of 20 LN patients and 20 SLE patients without LN, and miRNA sequencing was performed. The distribution characteristics and Venn analyses of miRNA revealed a difference between LN and SLE without LN (**Figures 3A, B**). In comparison to SLE patients without LN, 382 upregulated and 350 downregulated miRNAs were observed in LN, which met the criteria for sequencing detection of log2 fold-change > 2 in scatter plot analysis (**Figure 3C**). As shown in **Figure 3D**, the top 10 upregulated miRNAs visualized via hierarchical clustering were investigated as candidate markers of LN.

### Validation of RNA sequencing by RT-qPCR in the training phase

**Figure 4:**
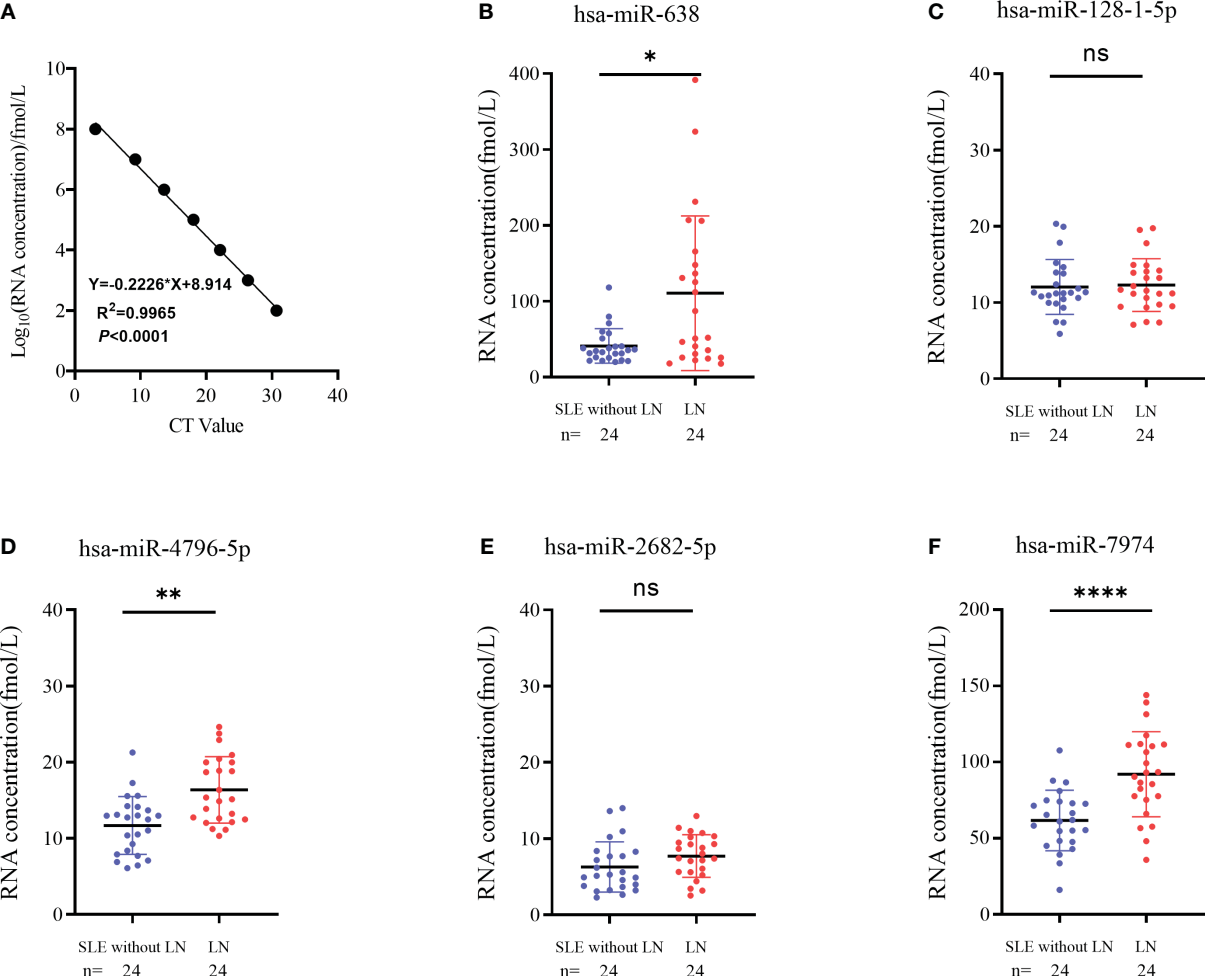
Identification of differentially expressed serum exosomes derived miRNAs in LNs and SLE without LN patients. **(A)** Linear standard curve of serum exosomes derived miRNAs concentration. **(B–F)** Differential expression of 5 miRNAs verified by RT-qPCR in LN and SLE without LN patients. hsa-miR-638, hsa-miR-4796-5p and hsa-miR-7974 were significantly upregulated in LNs compared with SLE without LN patients. *P* value of the Mann-Whitney U test: (*P < 0.05, **P < 0.01, ****P < 0.0001; ns, no significant difference).

To verify the results of sequencing, a total of 24 pairs of subjects (24 LN and 24 SLE without LN patients) were recruited in the training phase, employing the RT-qPCR method. To assess the reliability and repeatability of this method, a standard curve of different concentrations of synthetic miRNA was constructed to determine whether there was a linear relationship between 1 pmol/L and 10 nmol/L (**Figure 4A**). Out of the top ten upregulated miRNAs, five miRNAs were successfully amplified using the RT-qPCR method (**Figures 4B–F**). According to **Figure 4**, the absolute quantitative results indicate a significant increase in three serum exosomal miRNAs (hsa-miR-638, hsa-miR-4796-5p, and hsa-miR-7974) in LN patients compared to SLE patients without LN.

### Validation of RNA sequencing by RT-qPCR in the validation phase

**Figure 5:**
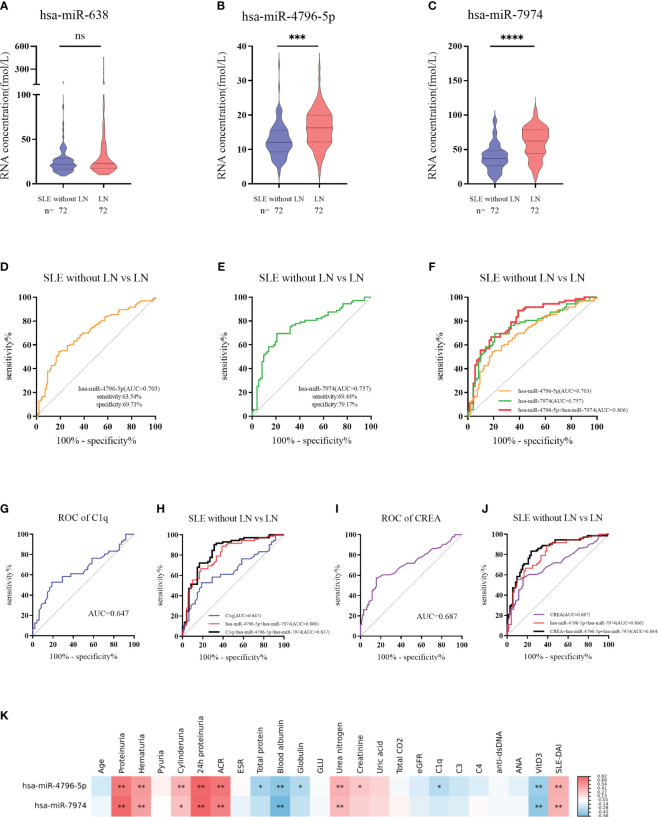
Diagnostic value of serum exosomes derived miRNAs in the validation phase. **(A–C)** Expression of 3 miRNAs in LNs compared with SLE without LN patients in large Samples. *P* value of the Mann-Whitney U test: (*P < 0.05, **P < 0.01, ***P < 0.001, ****P < 0.0001; ns, no significant difference) **(D)** Receiver operator characteristic (ROC) curve of hsa-miR-4796-5p in distinguishing LNs from SLE without LN patients. **(E)** Receiver operator characteristic (ROC) curve of hsa-miR-7974 in distinguishing LNs from SLE without LN patients. **(F)** ROC combined diagnostic analyses of hsa-miR-4796-5p and hsa-miR-7974 in discriminating LNs from SLE without LN patients. **(G, H)** ROC curve analysis of a single C1q marker and the combination of C1q with hsa-miR-4796-5p and hsa-miR-7974 markers for distinguishing LNs from SLE without LN patients. **(I, J)** ROC curve analysis of a single CREA marker and the combination of CREA with hsa-miR-4796-5p and hsa-miR-7974 markers for distinguishing LNs from SLE without LN patients. **(K)** Correlation analyses between miRNAs and clinical variables. Red and blue color represent the positive correlation and negative correlation, and the depth of the color represents the degree of correlation. The presence of * indicates an absolute value of the correlation coefficient r >0.3 (*P < 0.05, **P < 0.01 Spearman rank correlation analysis).

To further verify the above results, 144 subjects (72 LN and 72 SLE without LN patients) were recruited in the validation phase. According to **Figures 5A-C**, hsa-miR-4796-5p and hsa-miR-7974 are significantly elevated in LN compared with SLE without LN, while there is no difference in the level of hsa-miR-638 between the two cohorts. The diagnostic performance of two miRNAs, hsa-miR-4796-5p and hsa-miR-7974, was evaluated using ROC analysis. The area under the curve (AUC) values for hsa-miR-4796-5p and hsa-miR-7974 were 0.703 (95% CI: 0.6287-0.7767) and 0.757 (95% CI: 0.6766-0.8373), respectively. These AUC values were obtained for distinguishing between LN and SLE patients without LN, as shown in **Figures 5D, E**. The AUC for the panel of these two miRNAs combined was 0.806 (95% CI: 0.7348 to 0.8775), as demonstrated in **Figure 5F**. Serological indicators, such as C1q and CREA, have been found to be closely associated with renal damage caused by SLE. However, our study suggests that their diagnostic value is limited. In order to improve the accuracy of predictions, we evaluated the combined use of these clinical indicators with a panel of two miRNAs. Encouragingly, the AUCs for the combined approach were 0.837 and 0.844, respectively (**Figures 5G–J**). Furthermore, our findings revealed a positive correlation between levels of hsa-miR-4796-5p and hsa-miR-7974 with markers such as 24-hour proteinuria, ACR, creatinine, urea nitrogen, and SLEDAI. Conversely, we observed a negative correlation with albumin (Alb), C1q, and vitamin D3 levels (**Figure 5K**, **Supplementary Table 2**).

### Significance of hsa-miR-4796-5p and hsa-miR-7974 in clinical practice

**Figure 6:**
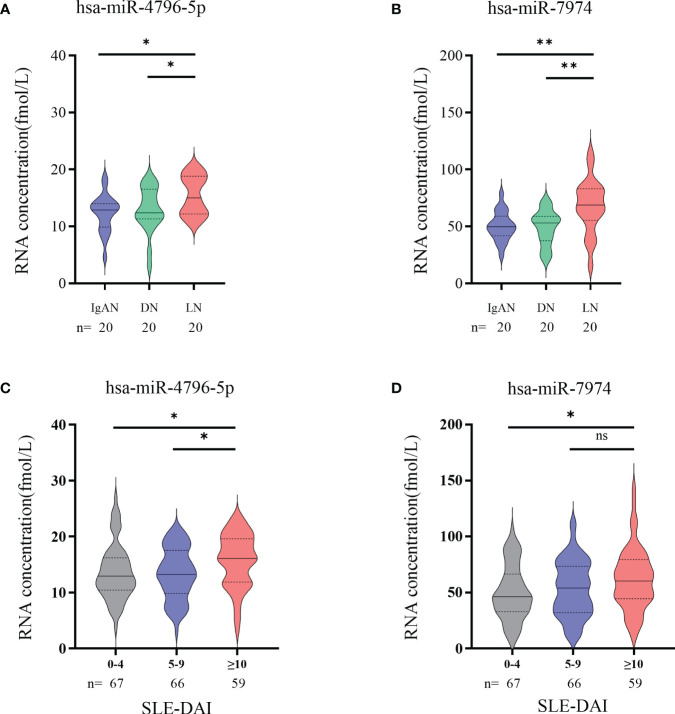
Significance of hsa-miR-4796-5p and hsa-miR-7974 in clinical practice. **(A, B)** Differential expression analysis of two miRNAs in different autoimmune nephritis diseases. Statistical significance was determined by the Mann-Whitney U test (*P < 0.05, **P < 0.01; ns, no significant difference). **(C, D)** Differential expression analysis of 2miRNAs in different LN severity groups. (0-4, no activity; 5-9, mild activity; ≥10, moderate to severe activity).

Autoimmune nephritis encompasses several types of kidney diseases, including immunoglobulin A nephropathy (IgAN), diabetic nephropathy (DN), anti-neutrophil cytoplasmic antibody (ANCA)-associated glomerulonephritis (GN), Henoch-Schonlein purpura nephritis (HSPN), and LN. In our hospital, the most prevalent types of autoimmune nephritis among patients are IgAN, DN, and LN. To investigate the specificity of miRNAs in diagnosing LN, we conducted experiments to evaluate the effectiveness of candidate exosomal miRNAs. A total of 20 patients with LN, 20 with IgAN, and 20 with DN were randomly enrolled in our study. Our findings revealed significantly higher levels of hsa-miR-4796-5p and hsa-miR-7974 in LN patients compared to those with IgAN and DN. (**Figures 6A, B**). Our findings reveal a noteworthy correlation between the levels of two specific miRNAs, hsa-miR-4796-5p and hsa-miR-7974, and the SLEDAI score. The correlation coefficients (r) and *p*-values for hsa-miR-4796-5p and hsa-miR-7974 are 0.423 (*p*=0.003) and 0.398 (*p*=0.005), respectively (**Supplementary Table S2**). Notably, the high scoring group exhibited a substantial disparity in miRNA levels when compared to the low scoring group. (**Figures 6C, D**).

### Enrichment analysis of hsa-miR-4796-5p and hsa-miR-7974

**Figure 7:**
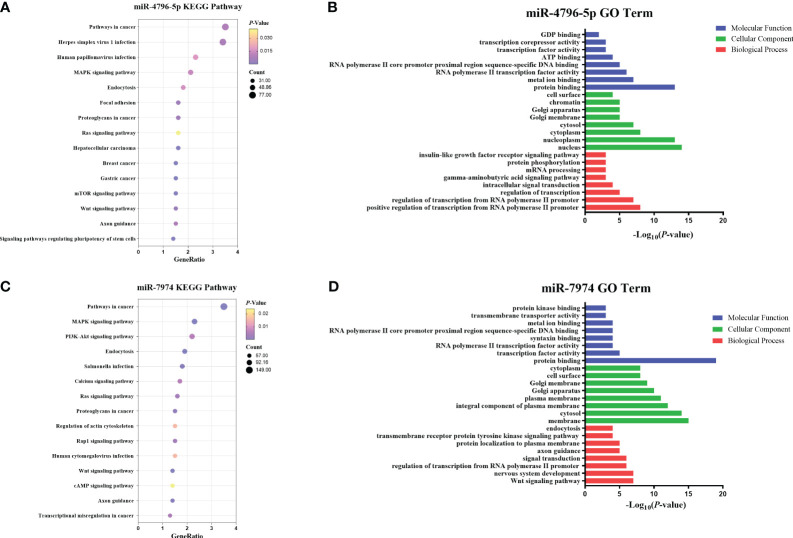
Enrichment analysis of hsa-miR-4796-5p and hsa-miR-7974. **(A, B)** KEGG pathway enrichment analysis and GO terms analysis of hsa-miR-4796-5p. **(C, D)** KEGG pathway enrichment analysis and GO terms analysis of hsa-miR-7974. The size of bubbles represents the number of genes enriched in this pathway, and the color of bubbles represents significance. P value represents the significance degree of enrichment.

To explore the potential functions of hsa-miR-4796-5p and hsa-miR-7974, we performed KEGG pathway enrichment analysis and Gene Ontology analysis. The KEGG results indicated that hsa-miR-4796-5p was associated with HSV-1 infection, the MAPK signaling pathway, and the mTOR signaling pathway (**Figure 7A**). The GO project of hsa-miR-4796-5p involves intracellular signal transduction, insulin-like growth factor receptor signaling pathway, protein phosphorylation, and other related processes (**Figure 7B**). KEGG pathway enrichment analysis of hsa-miR-7974 revealed the involvement of miRNAs in the MAPK pathway, PI3K-Akt signaling pathway, and endocytosis (**Figure 7C**). The analysis also identified enrichment of GO terms targeted by hsa-miR-7974, such as cellular responses to signal transduction, endocytosis, protein trafficking, and other GO strains (**Figure 7D**). These results serve as a reminder that miRNA may play an important role in the development of LN by impacting these functions.

## Discussion

LN is a severe manifestation of SLE, with approximately 40% of patients developing chronic kidney disease and 5-20% progressing to end-stage kidney disease (ESKD) within 10 years of their initial SLE diagnosis (32). Additionally, patients undergoing immunosuppressive treatment for LN may experience various complications such as infection, osteoporosis, cardiovascular issues, and reproductive system problems (4). Clearly, timely diagnosis and accurate evaluation of LN are essential for enhancing outcomes in SLE patients. The commonly used diagnostic methods for LN in clinical practice include 24-hour proteinuria quantification and kidney biopsy. However, these methods have certain limitations. Urine samples may have inaccurate retention time, and there may be poor patient compliance for urine protein testing. Additionally, kidney biopsy, although considered a valuable diagnostic tool, is an invasive procedure that carries the risk of bleeding and is challenging to replicate. Consequently, there is an immediate priority to investigate new non-invasive biomarkers that can effectively differentiate between LN and SLE.

Liquid biopsy is an innovative diagnostic method for analyzing biological material in blood and other bodily fluids to identify disease status (33). In recent years, the detection of miRNAs has been increasingly utilized for studying various autoimmune diseases. MiRNA, a small single-stranded endogenous non-coding RNA, has the remarkable capability to efficiently and precisely suppress the expression of its targeted transcripts, leading to alterations in cellular epigenetics and playing a vital regulatory role in both the innate and adaptive immune systems (34, 35). Current research on miRNAs in SLE primarily focuses on serum miRNAs and PBMC miRNAs, with limited studies on exosomal miRNAs (36, 37). Exosomes, a specific type of extracellular vesicles (EVs), have been identified in majority of body fluids (38–42). Composed of a lipid bilayer, the extracellular surface of exosomes serves as a protective barrier, safeguarding their contents such as proteins, mRNA, miRNAs, and other non-coding RNAs (ncRNAs) from degradation (18). When exosomes circulate, the RNA molecules they contain, especially miRNA, play a vital role in facilitating communication between different tissues through paracrine and endocrine pathways (43). Due to their excellent stability and accessibility, exosomal miRNAs hold potential as non-invasive biomarkers (44).

Our previous research has identified upregulated tsRNAs in the urine exosomes of patients with LN, which have shown promise in distinguishing LN from SLE (45). In this study, we aimed to analyze the levels of circulating exosomal miRNAs in serum to investigate their potential significance in LN. Previous reports have suggested that there may be differences in the miRNA content between blood plasma and serum (46, 47). Liu et al. (48) recommended the use of blood plasma in exosome research, as platelets also contain significant amounts of RNA that could be released into the serum during the coagulation process. However, a recent study (49) discovered that plasma prepared by centrifugation contains platelets and ery-ghosts, which co-isolate with EVs. In this study, we initially conducted RNA sequencing using serum exosomes from patients with LN. We compared the results to SLE patients without LN and identified 382 upregulated miRNAs in LN. Then, we selected the top 10 upregulated miRNAs as potential markers of LN. To validate the expression levels of these candidate exosomal-miRNAs, we used an RT-qPCR assay, which is more sensitive and not limited by sequence-abundance bias compared to the microarray profiling assay (50). Our data demonstrated that the levels of exosomal hsa-miR-4796-5p and hsa-miR-7974 were significantly elevated in patients with LN compared to SLE patients without LN in both the training and validation phases. These two miRNAs demonstrated a significant ability in diagnosing LN in patients with SLE, with an Area Under the Curve (AUC) above 0.8. In order to demonstrate higher diagnostic value, we formed a comprehensive team to integrate these two miRNAs with clinical parameters (such as C1q and CREA). The area under curve (AUC) values for these miRNAs were found to be 0.837 and 0.844, respectively. The SLE Disease Activity Index (SLEDAI) is an important tool for evaluating SLE activity based on clinical symptoms and auxiliary examinations. After scoring the 192 enrolled patients, we observed a significant upregulation of these two miRNAs in moderate to severe cases. Therefore, the exosomal hsa-miR-4796-5p and hsa-miR-7974 in serum have the potential to serve as biomarkers for evaluating disease activity.

In order to assess the specificity of these miRNAs duo in LN, we conducted separate experiments in immunoglobulin A nephropathy (IgAN) and diabetic nephropathy (DN). Our findings revealed that the levels of these two miRNAs were significantly elevated in LN compared to IgAN and DN. Therefore, these miRNAs not only serve as potential biomarkers to distinguish LN in SLE, but also exhibit good specificity in other autoimmune kidney diseases. The KEGG results revealed that hsa-miR-4796-5p and hsa-miR-7974 were found to be associated with various signaling pathways, such as the mTOR signaling pathway and PI3K-Akt signaling pathway which is implicated in the pathogenesis of LN (51–53). GO analysis showed that hsa-miR-4796-5p is enriched in insulin-like growth factor binding, which influences autoimmunity by modulating signaling pathways relevant to Th17/Treg balance (54). These results suggests that miRNAs investigated in this study may play a regulatory role in the progression of LN by engaging in these signaling pathways.

To recap, we have identified two serum exosomal miRNAs signatures in patients with LN. Specifically, we have demonstrated that hsa-miR-4796-5p and hsa-miR-7974, which are derived from serum exosomes, have the potential to be valuable biomarkers for differentiating between LN and SLE patients, as well as the autoimmune nephritis group. Furthermore, our study has shed light on the potential biological functions of these novel serum exosomal miRNAs. These findings provide a foundation for future research to explore the clinical applications and deeper understanding of serum miRNAs. However, it is important to note that our study was limited by the small sample size and single-center experience. Therefore, randomized clinical trials are the next frontier in evaluating these two miRNA signatures for LN diagnosis and prognosis.

## Data availability statement 

The data presented in the study are deposited in the NCBI repository, accession number PRJNA745976.

## Ethics statement

The studies involving humans were approved by The Ethics Committee of the Affiliated Drum Tower Hospital of Nanjing University Medical School. The studies were conducted in accordance with the local legislation and institutional requirements. The participants provided their written informed consent to participate in this study.

## Author contributions

FC: Data curation, Formal Analysis, Investigation, Methodology, Software, Writing – original draft. BS: Conceptualization, Data curation, Investigation, Methodology, Writing – original draft. WL: Conceptualization, Data curation, Investigation, Methodology, Software, Writing – original draft. JGo: Data curation, Methodology, Writing – original draft. JGa: Data curation, Investigation, Methodology, Writing – original draft. YS: Methodology, Writing – original draft. PY: Funding acquisition, Project administration, Supervision, Writing – review & editing. ZL: Funding acquisition, Resources, Supervision, Conceptualization, Writing – review & editing.

## Supplementary material

Supplementary Table 1 Specific primer information for 10 candidate miRNAs.

Supplementary Table 2 Correlation analysis between levels of hsa-miR-4796-5p and hsa-miR-7974 with laboratory markers and SLE-DAI. ACR, albumin-to-creatinine ratio; ESR, erythrocyte sedimentation rate; GLU, glucose; CREA, serum creatinine; eGFR, glomerular filtration rate; C1q, complement 1q; C3, complement C3; C4, complement C4; anti-dsDNA, anti-double stranded DNA antibody; ANA, antinuclear antibodies; SLE-DAI, systemic lupus erythematosus disease activity index.
