# Supplementary material for: Circulating exosomal microRNAs as biomarkers of lupus nephritis
Source: Front Immunol. 2023 Dec 29;14:1326836. doi: 10.3389/fimmu.2023.1326836 (PMC10785001; doi:10.3389/fimmu.2023.1326836)
Supplement: Supplementary Table 1 — Specific primer information for 10 candidate miRNAs. [file Table_1.doc]

**Supplementary Table S1: Specific primer information for 10 candidate miRNAs**

|  |  |  |
| --- | --- | --- |
| **miRNA** | **Primer sequence** | **Fold_Change** |
| hsa-miR-638 | RT: GTCGTATCCAGTGCAGGGTCCGAGGTATTCGCACTGGATACGACAGGCCG | 538.740019 |
|  | Q: GATCGCGGGCGGGTGG |  |
| hsa-miR-128-1-5p | RT: GTCGTATCCAGTGCAGGGTCCGAGGTATTCGCACTGGATACGACTCTCAG | 243.510204 |
|  | Q: CGGGGCCGTAGCACTGT |  |
| hsa-miR-512-3p | RT: GTCGTATCCAGTGCAGGGTCCGAGGTATTCGCACTGGATACGACGACCTC | 190.790595 |
|  | Q: CGCGAAGTGCTGTCATAGCT |  |
| hsa-miR-4796-5p | RT: GTCGTATCCAGTGCAGGGTCCGAGGTATTCGCACTGGATACGACGTAAAG | 159.158829 |
|  | Q: CGCGCGTGTCTATACTCTGTCA |  |
| hsa-miR-129-2-3p | RT: GTCGTATCCAGTGCAGGGTCCGAGGTATTCGCACTGGATACGACATGCTT | 159.158829 |
|  | Q: CGAAGCCCTTACCCCAAA |  |
| hsa-miR-2682-5p | RT: GTCGTATCCAGTGCAGGGTCCGAGGTATTCGCACTGGATACGACGACGTC | 148.614907 |
|  | Q: CGCAGGCAGTGACTGTTCA |  |
| hsa-miR-6746-3p | RT: GTCGTATCCAGTGCAGGGTCCGAGGTATTCGCACTGGATACGACCTGTGG | 138.070985 |
|  | Q: CAGCCGCCGCCTGTCT |  |
| hsa-miR-17-3p | RT: GTCGTATCCAGTGCAGGGTCCGAGGTATTCGCACTGGATACGACCTACAA | 138.070985 |
|  | Q: GCGACTGCAGTGAAGGCAC |  |
| hsa-miR-522-3p | RT: GTCGTATCCAGTGCAGGGTCCGAGGTATTCGCACTGGATACGACACACTC | 138.070985 |
|  | Q: CGCGAAAATGGTTCCCTTTA |  |
| hsa-miR-7974 | RT: GTCGTATCCAGTGCAGGGTCCGAGGTATTCGCACTGGATACGACGGGCTC | 127.527063 |
|  | Q: CGAGGCTGTGATGCTCTCCT |  |
|  |  |  |
