# Supplementary material for: Circulating exosomal microRNAs as biomarkers of lupus nephritis
Source: Front Immunol. 2023 Dec 29;14:1326836. doi: 10.3389/fimmu.2023.1326836 (PMC10785001; doi:10.3389/fimmu.2023.1326836)
Supplement: Supplementary Table 2 — Correlation analysis between levels of hsa-miR-4796-5p and hsa-miR-7974 with laboratory markers and SLE-DAI. ACR, albumin-to-creatinine ratio; ESR, erythrocyte sedimentation rate; GLU, glucose; CREA, serum creatinine; eGFR, glomerular filtration rate; C1q, complement 1q; C3, complement C3; C4, complement C4; anti-dsDNA, anti-double stranded DNA antibody; ANA, antinuclear antibodies; SLE-DAI, systemic lupus erythematosus disease activity index. [file Table_2.doc]

**Supplementary Table 2:** Correlation analysis between levels of hsa-miR-4796-5p and hsa-miR-7974 with laboratory markers and SLE-DAI.

|  | hsa-miR-4796-5p | | hsa-miR-7974 | |
| --- | --- | --- | --- | --- |
|  | r | *p* | r | *p* |
| Age | -0.178 | 0.226 | -0.076 | 0.610 |
| Proteinuria | 0.701 | 0.001 | 0.799 | 0.001 |
| Hematuria | 0.530 | 0.001 | 0.505 | 0.001 |
| Pyuria | 0.084 | 0.569 | 0.012 | 0.935 |
| Cylinderuria | 0.397 | 0.005 | 0.326 | 0.024 |
| 24h proteinuria | 0.802 | 0.001 | 0.818 | 0.001 |
| ACR | 0.712 | 0.001 | 0.656 | 0.001 |
| ESR | 0.034 | 0.821 | -0.105 | 0.477 |
| Total protein | -0.352 | 0.014 | -0.244 | 0.095 |
| Blood albumin | -0.453 | 0.001 | -0.555 | 0.001 |
| Globulin | -0.303 | 0.036 | -0.240 | 0.100 |
| GLU | -0.029 | 0.847 | -0.044 | 0.769 |
| Urea nitrogen | 0.402 | 0.005 | 0.372 | 0.009 |
| Creatinine | 0.295 | 0.042 | 0.242 | 0.097 |
| Uric acid | 0.222 | 0.129 | 0.198 | 0.177 |
| Total CO2 | -0.055 | 0.710 | 0.069 | 0.640 |
| eGFR | -0.155 | 0.293 | -0.120 | 0.416 |
| C1q | -0.301 | 0.047 | -0.158 | 0.282 |
| C3 | -0.209 | 0.154 | -0.147 | 0.319 |
| C4 | -0.145 | 0.325 | 0.033 | 0.823 |
| anti-dsDNA | -0.047 | 0.753 | -0.048 | 0.748 |
| ANA | -0.120 | 0.418 | -0.049 | 0.741 |
| VitD3 | -0.392 | 0.006 | -0.435 | 0.002 |
| SLE-DAI | 0.423 | 0.003 | 0.398 | 0.005 |

The correlation coefficients exceeding 0.3 are shown in gray color (Spearman rank correlation analysis).

Abbreviations: ACR, albumin-to-creatinine ratio; ESR, erythrocyte sedimentation rate; GLU, glucose; CREA, serum creatinine; eGFR, glomerular filtration rate; C1q, complement 1q; C3, complement C3; C4, complement C4; anti-dsDNA, anti-double stranded DNA antibody; ANA, antinuclear antibodies; SLE-DAI, systemic lupus erythematosus disease activity index.
